# Conceptualizing the impacts of dual practice on the retention of public sector specialists - evidence from South Africa

**DOI:** 10.1186/1478-4491-13-3

**Published:** 2015-01-19

**Authors:** John Ashmore, Lucy Gilson

**Affiliations:** Health Economics Unit, School of Public Health and Family Medicine, Falmouth Annex, Medical Campus, University of Cape Town, Observatory, Cape Town, South Africa; Department of Global Health and Development, London School of Hygiene and Tropical Medicine, Keppel Street, London, England

**Keywords:** Multiple job holding, Public private mix, Retention, Migration, Loyalty, Health worker distribution

## Abstract

**Background:**

‘Dual practice’, or multiple job holding, generally involves public sector-based health workers taking additional work in the private sector. This form of the practice is purported to help retain public health care workers in low and middle-income countries’ public sectors through additional wage incentives. There has been little conceptual or empirical development of the relationship between dual practice and retention.

**Methods:**

This article helps begin to fill this gap, drawing on empirical evidence from a qualitative study focusing on South African specialists. Fifty-one repeat, in-depth interviews were carried out with 28 doctors (predominantly specialists) with more than one job, in one public and one private urban hospital.

**Results:**

Findings suggest dual practice can impact both positively and negatively on specialists’ intention to stay in the public sector. This is through multiple conceptual channels including those previously identified in the literature such as dual practice acting as a ‘stepping stone’ to private practice by reducing migration costs. Dual practice can also lead specialists to re-evaluate how they compare public and private jobs, and to overworking which can expedite decisions on whether to stay in the public sector or leave. Numerous respondents undertook dual practice without official permission.

**Conclusions:**

The idea that dual practice helps retain public specialists in South Africa may be overstated. Yet banning the practice may be ineffective, given many undertake it without permission in any case. Regulation should be better enforced to ensure dual practice is not abused. The conceptual framework developed in this article could form a basis for further qualitative and quantitative inquiry.

**Electronic supplementary material:**

The online version of this article (doi:10.1186/1478-4491-13-3) contains supplementary material, which is available to authorized users.

## Background

‘Dual practice’ refers to health workers holding multiple jobs of various kinds [1–3]. This article, however, along with others in the dual practice literature [4, 5], focuses only on dual practice in the form of public-based health workers working part-time in the private, for-profit sector, either within the same facility or outside of it (termed ‘public on private’ dual practice by Ferrinho and colleagues [2]).

Little is known about this practice, despite its commonality in many countries [6]. There is a substantial lack of evidence on the impacts of dual practice, especially in low- and middle-income countries (LMICs). This is problematic given there may be benefits to dual practice which regulation might encourage, and costs which regulation might curtail [7, 8]. The only real consensus in the literature so far is that dual practice carries both costs and benefits, and that more research is needed to understand these costs and benefits in specific contexts. It is also universally noted that, in terms of regulation, dual practice is difficult to ban [4, 9, 10].

One important set of impacts demanding further attention can be termed ‘retention impacts’. These refer to how dual practice affects the likelihood of health workers staying in the public sector, as opposed to moving into private practice or even leaving their countries.

Much of the scant literature in this area asserts that LMIC health workers are more likely to stay in the public sector as a result of dual practice, since it is often undertaken as a ‘coping’ or income-generating strategy. Health workers’ income expectations often go unmet in LMIC public sectors alone [11–13]. This argument provides a rationale for allowing dual practice, to bolster health worker capacities in the public sector where they are most needed. As Bir and Eggleston [14] put it, ‘since skilled clinicians expect to be able to generate significant income from private practice, allowing dual practice may be one of the most effective policies that a cash-strapped low-income government can use to retain skilled physicians in public clinics and hospitals'.

Conversely, Chikanda [15] speculates that dual practice may act as a ‘stepping stone’ to leaving the public sector, in that health workers could use additional private income to cover public-to-private or international migration costs. Others have shown dual practice to be associated with some absenteeism from the public sector [12, 14]. All this implies dual practice may exacerbate inequities in health worker distribution, internationally and within LMICs.

There is very scarce conceptual development in terms of channels of influence between dual practice and retention. This article aims to help fill this gap by unpacking the retention impacts of dual practice in a specific LMIC setting where the practice is very common.

Qualitative evidence is presented from South Africa, a highly unequal middle-income country where retaining public health workers is an explicit priority of the National Department of Health [16]. This priority results from the country’s inequitable ‘two tier’ health system, divided along public-private lines [17]. Around half of all GPs and specialists, and 30% of nurses, work in the private sector [18], serving the richest 15% of the population with health insurance [19]. Those without health insurance have acute health care needs. Household data estimates the poorest 80% of the population carries around 94% of the HIV burden, and 100% of the tuberculosis (TB) burden [20], for example. In combination, HIV and TB constitute the largest disease burden in South Africa [21].

South Africa was also an appropriate study location since public health workers have been permitted to work part-time in the private sector since the early 1990s, initially through the Limited Private Practice policy and, since 2001, through the policy of ‘RWOPS’ (Remunerated Work Outside the Public Service). Limited Private Practice was introduced to help retain public health workers [22, 23], yet there is no evidence to suggest this was effective (or ineffective). Concerns have subsequently been raised about RWOPS, in terms of greater absenteeism resulting from diversion of public time to private activities [24]. This paper is limited only to issues of retention of health workers, however, rather than other impacts such as absenteeism.

The study undertaken for this paper focused on doctors, and in particular, specialists. This is since doctors appear most likely, in any setting, to undertake dual practice out of all health workers [2, 6]. In South Africa, dual practice has also previously been asserted as particularly common among specialists [25].

The article now outlines the initial organization theory framing of the study before proceeding. A conceptual framework is developed later after empirical evidence is presented. The conceptual framework development in this article provides additional insights to the previous Ashmore [26] paper which focused only on job satisfaction comparisons between public and private sectors. This is since, unlike the previous paper, this article specifically addresses one set of impacts of dual practice itself, rather than a general comparison of public and private sectors. It also goes considerably beyond the conceptual framing of ‘job satisfaction’ as a predictor of retention to evaluate other intermediary predictors in the context of dual practice. The paper could be considered an intermediary study within health policy and systems research, in terms of incrementally drawing on both empirical and conceptual evidence for the purposes of bolstering future studies.

### Conceptual framing

Organization theory, the theoretical base of this paper, draws on fields as wide as psychology, sociology and anthropology, as well as various theories of economics, to explain organizational functioning and behaviour [27]. In organization theory, the concepts of ‘motivation’, ‘job satisfaction’ and ‘loyalty’ are commonly used to understand a variety of workplace decisions. Motivation, adopted by some researchers in the health systems literature [28, 29], refers to an, '*individual’s degree of willingness to exert and maintain an effort towards organizational goals*' [30]. Motivation is an aspirational, goal-oriented concept.

The concept of job satisfaction has also been practically applied elsewhere in the health systems literature [31, 32]. Locke [33] defines job satisfaction as '*a pleasurable or positive emotional state resulting from the appraisal of one’s job or job experiences*'. Job satisfaction factors are noted by Spector’s [34] review of the literature to commonly fall into the following four groupings: 1) financial rewards, such as income and benefits; 2) organizational context, including resource availability; 3) other people, including satisfaction with patients and colleagues; and 4) the nature of the work itself, such as how mentally stimulating the work is and whether it is varied or monotonous. Both job satisfaction and motivation can have similar determinants, relating to the fulfilment of needs, values and expectations, but job satisfaction is more empirically and conceptually related to retention whereas motivation is more performance-related [29, 32, 35].

Ashmore [26] previously used Spector’s [34] categorization of the concept of job satisfaction to expand upon reasons for South African specialists choosing to work in the public versus private sector and often both. The present article similarly takes job satisfaction as a conceptual starting point and useful way to understand the way dual practice affects intention to stay in the public sector. This is since, here, ‘job satisfaction’ is assumed to increase through dual practice, through the provision of additional incentives available in private health sector work. Jobs can, in this light, be seen as complementary rather than substitutable. This, in turn, is assumed to increase intention to stay in the public sector in much the same way as intention to stay would increase through higher ‘utility’ in economic theory. Previous studies have found strong and significant correlations between job satisfaction, intention to stay and actual retention behaviour [36].

The concept of ‘organizational loyalty’, meanwhile, is herein defined as *commitment to a workplace, the people in the workplace, and/or the work itself* [33]. It is assumed to mediate or control incentives to leave the public sector. Loyalty has been found to affect retention where job satisfaction is already low (see meta-analyses [36, 37]). One would also expect loyalty to mediate any incentives to use dual practice as a 'stepping stone' into the private sector, since it is understood in the literature as a retention-enhancing concept in general.

Importantly, however, job satisfaction and loyalty are but two parts of a more complex staying/leaving decision process [33, 34]. Mobley [38] expanded upon this through his review of the organizational literature. They constructed a model where an employee’s evaluation of their job, based around their expectations and values being (un)met, leads them to be (dis)satisfied. Mobley stressed that workers then evaluate the costs and benefits of quitting, search for and compare alternative job options, and only then decide to stay or leave. There is also the possibility that workers act ‘impulsively’ about retention decisions. These latter steps in the causative model, impulsive or not, occur after the variable ‘intention to stay’. Intention to stay is the most proximate predictor of retention this paper deals with, since retention itself could not be observed during research due to the cross-sectional and self-reported nature of the qualitative study.

It is important to emphasize that the above concepts, later used to understand the relationship between dual practice and retention in a specific empiric setting, assume that both financial and non-financial incentives affect retention. This is since there is evidence in the literature to suggest dual practice may be satisfying by providing non-financial, in addition to financial, incentives. Humphrey and Russell’s [39] qualitative study found UK specialists valued additional jobs for ‘greater strategic influence and clinical autonomy, a greater sense of being valued, and more opportunities to realize … individual aspirations as clinicians’. Reasons for dual practice mentioned by a variety of New Zealand health workers, meanwhile, often centred around increased ‘variety’, ‘enjoyment’, and ‘challenge’ of additional work, rather than just money [40].

## Methods

In-depth, conversational interviews were conducted in an anonymous South African city and were helpful to derive conceptual channels of influence between dual practice and retention. Interviews were conducted in two field sites: one large public academic hospital, and one large private hospital - located close to one another. Hospitals were anonymized as ‘H1’ and ‘P1’, respectively, to protect respondents’ identities. Twenty-three semi-structured interviews were conducted with key informants in the same city as the hospitals, for background, including doctors who did not undertake dual practice, hospital managers and provincial policy-makers. Key informants were asked their views on dual practice, what forms it generally takes, and also acted as gatekeepers.

The ‘core’ interview sample included twenty-eight doctors in H1 and P1 with more than one job of one type or another - predominantly RWOPS. The majority of respondents were specialists (n = 20/28) or specialists in training (n = 7/28), known as ‘registrars’. This reflected the fact that among doctors it was mostly specialists who undertook RWOPS in and around H1 (as confirmed by key informants): in fact, only specialists were allowed to. The core sample was located by inviting participants through departmental e-mail lists, and through a snowballing approach. Interviewing ceased once ‘saturation point’ was reached, that is once no new major codes were arrived at after three consecutive interviews [41].

Respondents were first asked open ended questions: ‘Can you tell me about your experience of holding more than one job?’, and, ‘What are your feelings about having more than one job?’ before probing further on hunches. Follow-up interviews asked respondents directly whether and how dual practice was understood to affect retention. The 51 total interviews with the core sample were recorded and transcribed to retain ‘thick’ context in the data [42]. Five respondents were not available for follow-up interviews, three of whom noted they were too busy and two of whom were not contactable.

Interviews were spread across six departments within the two hospitals (alphabetically: anaesthesiology, medicine, neonatology, obstetrics and gynaecology, psychiatry, and surgery). Respondents’ ages ranged from 29 to 63, and 36% (10/28) of respondents were women. Although only 11% (3/28) of respondents were black, this reflected a tendency for dual practice doctors working in the urban hospital in question to be specialists, and for specialists in South Africa to be white [43]. Repeated, unsuccessful attempts were made to track down further black respondents including through snowballing (asking respondents if they know of other, sought-after respondents).

Analysis was performed through ‘memoing’ (inductively building up broad, explanatory hypotheses as interviews progressed), and later collating these memos to derive coding categories. Coding was performed in Microsoft OneNote (Microsoft, Redmond, WA, USA) using its categorization capabilities and through building ‘hyperlink’ lists linking to relevant paragraphs of transcribed data. There was a back-and-forth learning process used to validate data - for example, where contradictory answers were given by different respondents, reference would be made to policy documents where understanding of policies conflicted. Key informant interviews also helped to triangulate answers. Questions during follow-up interviews also probed into what prior respondents had said and how different individuals interpreted certain points of view.

## Results

Additional file 1 summarizes the hierarchy of hospital-based doctors in South Africa for those unfamiliar with the context. In practice, the private work undertaken by specialists in South Africa is often undertaken in private wards established in the public sector [44]. Interview respondents also sometimes had their own private rooms, meaning an established private practice; or covered other specialists on private locums, the latter generally implying temporary private work. Some specialties were noted to be more profitable in terms of dual practice, and dual practice appeared popular in these disciplines. In particular, surgery and anaesthesiology were said to pay very well in the private sector.

RWOPS policy is subject to provincial government interpretation. RWOPS was viewed by policy-makers interviewed at provincial level as a ‘privilege’ and not a ‘right’, meaning it could be withdrawn at any point. Around the time of fieldwork, in fact, RWOPS was entirely banned in another of South Africa’s nine provinces.

To apply for RWOPS where fieldwork was conducted, individuals had to justify how their practice was beneficial to the government and how they would spend their time on a weekly basis. Seven respondents interviewed had never applied for RWOPS permission. H1’s hospital CEO and other key informants noted that in spite of RWOPS policy allowing for asset seizure and prosecution for fraud if RWOPS was not applied for or public sector duties were neglected, respectively, these threats had never have been carried out in H1 (see Additional file 2 for details on the specifics of RWOPS policy at the time of fieldwork).

Oversight of dual practice varied by department in H1. One department actively encouraged RWOPS through an on-site private wing in the public hospital, saying everyone needed to ‘chip in’, and regularly checked rotors for deviation. Revenues from this private work were shared equally among the specialists in the department. Another department banned RWOPS outright, though several came forward in this department who undertook private work without permission. A third department took a very *laissez-faire* approach, where two respondents were interviewed who appeared to fall under legal definitions of fraud. In terms of locum work, which was relatively rare for specialists interviewed, a private sector administrator was interviewed who admitted to actively recruiting registrars who are not allowed to undertake dual practice at all since they are still in training. Some respondents noted that some medical officers (more junior doctors) also undertake locums regularly though this is not allowed in H1. No such medical officers came forward for interview after repeated invitations, though some specialists said they previously undertook locums when they were Medical Officers.

### Positive impacts of dual practice on intention to stay

Qualitative interviews indicated dual practice could positively affect intention to stay in the public sector for some specialists by increasing job satisfaction. In interviews, dual practice was generally perceived as an inherently satisfying endeavor for those who chose to undertake it, explaining both why it was sustained over time, and why it can increase intention to stay in the public sector. Ashmore [26] previously presented a variety of financial and non-financial factors that dual practice specialists valued in their public and private jobs. These factors included, for example, that a number of specialists enjoyed balancing the better wages, greater autonomy, and higher resource availability in the private sector, with the collegiality, greater variety of teaching and research work, and more complex pathology in the public sector [26].

Additional interview evidence further demonstrates that dual practice respondents *explicitly* drew the link themselves, between holding multiple jobs, job satisfaction, and intention to stay in the public sector:

*Ja*, I think overall it’s (RWOPS) probably the thing that keeps me in my government job actually, because with my skills and my training I don’t feel I get paid what I should get paid, but I enjoy working here (in H1), so that’s the most important thing for me … I think with the combination of private work and (public work) … I’m able to supplement my income, so it’s quite a good income now and getting towards a competitive income compared with if I was to work in private. (H1 DP17, Interview 1)

And I think obviously the other thing that is central to the private practice thing is that you’re likely to make more money out of it … Which is the core aspect of why I do it, and why pretty much everybody else does it … because it makes your salary a little bit more competitive with what your alternatives are somewhere else. (H1 DP7, Interview 1)

*Ja*, I think … (RWOPS) does (help retain me) because you get frustrated if you feel you don’t get enough operating time (in public), you don’t get enough time to do (specific surgical procedures) and you can’t … see how you’re going to make it work for you. For me I think it (RWOPS) is a positive experience. (H1 DP14, Interview 2)

The first two extracts above speak to financial job satisfaction factors associated with dual practice that can be retention-enhancing, while the third speaks to a non-financial job satisfaction factor having a similar effect. The majority also agreed in follow-up interviews that they simply enjoy RWOPS, ‘otherwise I wouldn’t do it’, implying self-selection into dual practice job arrangements.

However, these data suggest that the concept of job satisfaction must itself be adjusted here to one of ‘work satisfaction’, meaning satisfaction in one or more jobs. This is since, in interviews, satisfaction seemed to change across multiple jobs as a result of dual practice, not just in one job. Work satisfaction seemed to spill over from one job to the next as a result of dual practice since public and private jobs offered unique incentives that led to jobs being complementary rather than substitutable. For example, several respondents noted they were able to keep their ‘hand in’ through RWOPS, meaning they were able to perform procedures in the private sector that only more junior doctors performed in public.

Not all specialists interviewed felt dual practice was entirely satisfying, however. Some highlighted opportunity costs or tradeoffs to the practice, through having less free time with friends and family and for other activities like exercise. This led to ‘strained personal relationships’ and compromised ‘work-life balance’:

… (overall satisfaction) increases (through RWOPS) because you get paid more which means you can afford nicer things I guess, and it increases because you enjoy the work component of your life more; but it decreases because you spend less time with your kids and you’re overworked. (H1 DP16, Interview 2)

This implies that while dual practice may tend to increase *work* satisfaction, it may by the same token decrease *life* satisfaction, that is satisfaction with life outside work. Overall, it appeared as if such opportunity costs were worthwhile, since specialists generally noted they would otherwise not be willing to continue with their multiple jobs. During follow-up interviews, all specialists noted either a positive or equivocal response when asked whether they felt dual practice was satisfying, overall, for work and life outside work. Some respondents noted their penchant for workaholism. A registrar explained this by saying, ‘doctors are funny … we always have this thing of proving ourselves … you try and test yourself in different situations to see whether you can cope with them'. Another respondent noted their personal work ethic boiled down to the phrase, ‘I work like hell’.

### Negative or equivocal impacts of dual practice on retention

This implies that even if dual practice is understood as satisfying overall, it may not prove sustainable in the long-run. Many in H1 noted they were extremely overworked, and that their extra job exacerbated this problem. The following private specialist, located in P1, explained that they had previously opted to leave H1, for full-time private work, after the dual practice arrangement proved too much to handle:

… I was working as a full-time consultant (in H1), and I was doing limited private work at (a private hospital) … and I was involved in teaching … I was doing three jobs. I was trying to supplement my income and provide a service to private patients. I was fulfilling my … commitments to service delivery and then trying to have an academic career … and that just wasn’t possible … (P1 DP3, Interview 1)

In this regard, dual practice appeared capable of expediting a decision on whether to stay in the public sector or not, bringing some respondents to a point where they felt ‘forced to choose’ between their public and private jobs. While the above respondent sided with the private sector, the following respondent chose to only work in the public sector after feeling overwhelmed by dual practice:

I decided at 0200 h one morning when I was doing a difficult (case) that I actually couldn’t keep all the balls in the air. And on one hand it was very nice earning extra money (through dual practice) - it just gives you that little more flexibility and one would be a liar to say it doesn’t; but on the other hand you also need to know what your priority is. And you know, I’ve got academic aspirations. (H1 DP15, Interview 1)

It was also noted that how specialists *compared* public and private jobs could change as a result of dual practice. This could also undermine intention to stay in the public sector. For example, the following respondent explained that although their work satisfaction increased through dual practice, their satisfaction with the public sector decreased, after they became frustrated with poorly functioning services:

… private work doesn’t add to public satisfaction. In fact it detracts a little bit because you get a bit frustrated with how things work in public. (H1 DP18, Interview 2)

Yet not all respondents felt this way. Others explained their private jobs made them more appreciative of the public sector, as with the following junior specialist:

Doing private locums makes me more want to stay in public sector … Sure you earn maybe … four or five times what you’re earning in the state, but I’m not sure it’s worth it. You stress, you have people phoning you all the time. It’s heavy, it’s the heaviest responsibility … (H1 DP18, Interview 1)

More worryingly, from a public sector retention perspective, interview respondents also mentioned dual practice could reduce migration costs to the private sector, thus facilitating public specialists moving to the private sector. Respondents noted, in particular, that the first few years of private practice could be relatively unprofitable, since ‘you spend a lot of time sitting in your office hoping that somebody is going to knock on the door’. Dual practice could help build access to a steady patient pool through referral networks of General Practitioners (GPs) prior to leaving the public sector. As several respondents agreed, this allowed specialists to use dual practice and RWOPS as a ‘stepping stone’ and to ‘launch careers’ in the private sector:

I think definitely people (use RWOPS to launch private careers) … I mean they have a salary here (in H1), and they need to build up a practice; and if you just go ‘out there’ (into private) immediately they have costs and things, so they tend to do RWOPS. (H1 DP2, Interview 2).

Initial cost outlays for private practice included building referral networks, as mentioned, but also other sunk costs including buying particular machinery, such as ultrasound machinery, and emotional costs associated with getting to know a new place, new people, and a new system. Not all specialists seemed to face all these problems, however. Some mentioned being invited to take over or join already existing practices.

In interviews, no respondents admitted to using dual practice as a 'stepping stone' explicitly (and it was felt inappropriate to directly press this question), but a number of individuals did mention others for whom they believed this to have been the case.

### Revised conceptual framework

Thus, it seems that dual practice provides both incentives to stay in the public sector, through increased overall work and life satisfaction (or ‘work-life satisfaction’), and incentives to move to the private sector, through reduced migration costs or ‘stepping stone’ effects. In particular, it was found that dual practice allowed specialists to build up GP referral networks while maintaining a full-time government wage, making moving permanently to the private sector less costly in the short-term than if they were to do so immediately. Additional insights not previously found in the literature include that dual practice may also affect retention by proving unsustainable through overworking, forcing a decisions on whether to stay in the public sector or ‘go private’ that might otherwise seem less imminent. Dual practice may also change the way public and private jobs are compared with one another, in terms of either feeling more frustrated with the public sector having seen how people work in private, or *vice versa*.

**Figure 1:**
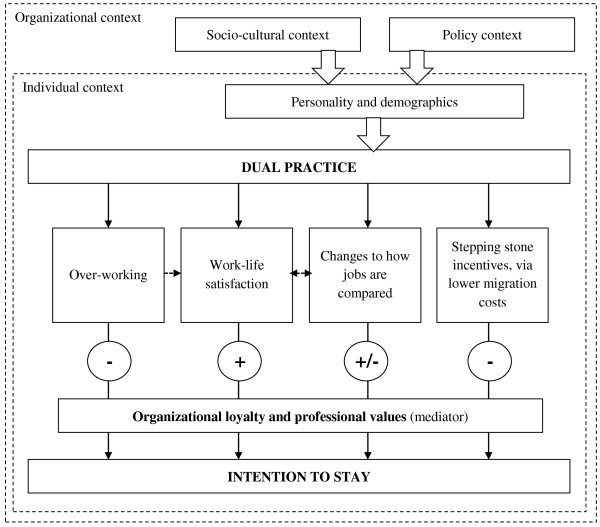
**Revised conceptualization of the relationship between dual practice and intention to stay in the public sector.**

This summary of conceptual channels between dual practice and intention to stay is depicted in Figure 1 below. The conceptual framework, similarly to Franco *et al*.’s [30] motivational framework in a health care context, assumes contextual factors (sociocultural, policy and personal, factors) filter down to the individual level, informing the particular context of dual practice in a specific setting. Such contextual factors are evident above in the particularities of, for example, individuals who were strongly encouraged to undertake dual practice by their own department (versus those not allowed to in another department) which may affect whether the practice is enjoyed and sustained. The importance of personal factors is also evident in the extracts from interviews with respondents H1 DP15 and P1 DP3 above. After feeling highly overworked, these two respondents chose to drop their private and public sector jobs, respectively, due to personal values held. Such contextual factors undoubtedly play a strong role in determining the dynamics surrounding whether dual practice helps or does not help to retain specialists in a specific setting.

In Figure 1, positive and negative channels for retention (or rather, intention to stay) are expressed with + and - signs. It is also important to note that the figure draws a connection between overworking and work-life satisfaction, as well as an interaction between changes in how jobs are compared and work-life satisfaction. The latter is since working in a second job may increase or decrease satisfaction in a primary job, while overworking appeared to cause general frustration.

Finally, it is important to discuss the issue of organizational loyalty. In the figure above, loyalty mediates all incentives to leave the public sector, but is not affected by dual practice. This is since, in follow-up interviews, respondents’ answers converged in terms of noting that their loyalties to the public sector were essentially formed earlier on in their careers; for example, through the influence of experiences in rural settings after graduating and through the influence of role models. Loyalty was generally noted to relate to public sector patients and colleagues, as well as to the work itself and personal or professional values, but infrequently to the public sector administration or Department of Health:

My loyalty is not to the province (provincial government), it’s loyalty to my own value system. For a lot of us it lies in serving the communities that need us most, and that really has nothing to do with the state who doesn’t serve them very well. It’s really in spite of the state, not because of them, that we work. (H1 DP6, Interview 2)

I think the most important thing is to create the right perception amongst your colleagues. To always make sure the conflict (between jobs) is kept to a minimum, and if there is going to be conflict that you default always towards your government work because that’s where your commitment should be. (H1 DP17, Interview 1)

While loyalty appeared predetermined, it nevertheless may be very important as a mediator to low work satisfaction, given some specialists continued to work in the public sector despite significant frustrations. Loyalty also appeared to mediate incentives to leave the public sector where dual practice arrangements became unsustainable through overworking, as in the figure above, in cases where private sectors jobs were dropped in favour of public sector jobs.

Loyalty also mediated incentives to use dual practice as a 'stepping stone' into private practice, given these incentives were often present but not always acted upon. While some individuals in H1 felt they were keeping their ‘options open’ through dual practice, others used the practice specifically as a means to stay in public practice.

## Discussion

The results and framework above elaborate both positive and negative channels of influence between dual practice and intention to stay in the public sector. The existence of these different channels implies the relationship between dual practice and retention is complex and context-dependent. Context dependency is implied in that different policy environments will likely provide different incentives to health workers, and allow different opportunities in public and private sectors. Even within the same country it is likely that dual practice will in different settings sometimes have positive, and sometimes negative, net impacts on retention.

In the research setting, the fact that many individuals found public sector hospital work and pathologies interesting may be one of the contextual reasons many were willing to stay on in the public sector. Where health workers are less academically inclined, or less inclined towards workaholism, this finding may differ. In addition, other contexts will not have on-site private sector work available to them, which may imply greater likelihood of individuals seeking dual practice in fully private hospitals. This may in fact be expected to increase the ‘stepping stone’ effects associated with building up a GP referral base, which could undermine retention in the public sector further.

The study was limited by only speaking to intention to stay, rather than observed retention behaviour, though as noted the two tend to correlate well. The specific context of the study also means additional conceptual insights might be found elsewhere, though overlapping themes would be expected and the conceptual framework may well be relevant beyond this South African hospital setting. The fact that the majority of specialists in this study were white and from a relatively limited cultural background also implies that additional channels of influence between dual practice and retention could be found in future studies.

Overall, some specialists appear to use dual practice as a means to stay in the public sector, through higher work-life satisfaction, in spite of opportunity costs to dual practice; others appear to feel dual practice is unsustainable over the long-run. Dual practice may also change how public and private job options are compared, and provide incentives to exit the public sector through reduced migration costs. Some of these channels of influence between dual practice and intention to stay were expected from the literature, though to these authors’ knowledge this paper represents the first structured conceptual inquiry into this area. Organizational loyalty was found to be an important contextual mediator to incentives to leave the public sector, and was found to be determined early on in doctors’ careers.

South African policy-makers should take note that RWOPS may not be working in the interests of boosting public sector retention, at least for specialists, and that the policy could well be failing in its aims. Importantly, however, this is not to say that banning RWOPS would necessarily be beneficial, since it is clear from other countries’ experiences [9], and from discussions during interviews in H1 and P1, that banning dual practice may well backfire. This is since specialists tend to view a ban on dual practice as threatening, and since dual practice often persists when banned [9, 45]. There are also other impacts of dual practice which this paper did not evaluate, such as absenteeism, which may be equally important to factor into an evaluation of dual practice costs and benefits.

As such, it seems that the primary lesson from this paper is that it is questionable whether dual practice increases retention in the public sector, and that banning the practice may be more questionable than strengthening accountability mechanisms such as careful roster monitoring. This may be particularly the case in surgery and anaesthesiology, for example, where roster systems are based around theatre times and where many undertake dual practice. It may also be beneficial to consider strengthening internal whistleblower mechanisms and how information about dual practice abuse is acted upon, given nobody in H1 had received any punishment for RWOPS abuses in spite of several clear examples.

Other studies may wish to quantitatively test the relationship between dual practice and retention by drawing on this conceptual framework, and to investigate whether the framework applies beyond the context of specialists and to other cadres of health workers. Health policy and systems research could learn from this study in terms of similarly using qualitative research to explore conceptual relationships in highly understudied areas.

## Electronic supplementary material

Additional file 1: **Hierarchy of South African hospital doctors.** (PDF 46 KB)

Additional file 2: **Summary of 2009/10 RWOPS regulations.** (PDF 142 KB)

## Abbreviations

GP: General Practitioner
HIV: Human Immunodeficiency Virus
LMIC: Low or Middle-Income Country
RWOPS: Remunerated Work Outside the Public Service
TB: Tuberculosis.

## References

[1] Berman P, Cuizon D (2004). Multiple public-private job holding of healthcare providers in developing countries: an exploration of theory and evidence.

[2] Ferrinho P, Lerberghe WV, Fronteira I, Hipólito F, Biscaia A (2004). Dual practice in the health sector: review of the evidence. Hum Resour Heal.

[3] Roenen C, Ferrinho P, Van Dormael M, Conceição MC, Van Lerberghe W (1997). How African doctors make ends meet: an exploration. Trop Med Int Health.

[4] Garcia-Prado A, Gonzalez P (2007). Policy and regulatory responses to dual practice in the health sector. Health Policy.

[5] Gruen R, Anwar R, Begum T, Killingsworth J, Normand C (2002). Dual job holding practitioners in Bangladesh: an exploration. Soc Sci Med.

[6] Gupta N, Dal PM (2009). Assessment of human resources for health using cross-national comparison of facility surveys in six countries. Hum Resour Heal.

[7] Jumpa M, Jan S, Mills A (2003). Dual practice of public sector health care providers in Peru. Health Economics and Financing Programme (HEFP) working paper 06/03.

[8] Jumpa M, Jan S, Mills A (2007). The role of regulation in influencing income-generating activities among public sector doctors in Peru. Hum Resour Heal.

[9] Kiwanuka S, Rutebemberwa E, Nalwadda C, Okui O, Sengooba F, Kinengyere A (2011). Interventions to manage dual practice. Cochrane Database Syst Rev.

[10] Socha K, Bech M (2010). Physician dual practice: a review of literature. Health Policy.

[11] Ferrinho P, Van Lerberghe W, Julien M, Fresta E, Gomes A, Dias F (1998). Research report. How and why public sector doctors engage in private practice in Portuguese-speaking African countries. Health Policy Plan.

[12] Macq J, Ferrinho P, De Brouwere V, Van Lerberghe W (2001). Managing health services in developing countries: between ethics of the civil servant and the need for moonlighting. Hum Resour Heal.

[13] Muula A, Maskeo F (2006). How are health professionals earning their living in Malawi?. BMC Health Serv Res.

[14] Bir A, Eggleston K (2003). Physician dual practice: access enhancement or demand inducement?.

[15] Chikanda A (2004). Skilled health professionals’ migration and its impact on health delivery in Zimbabwe.

[16] National Department of Health of South Africa (2011). Human resources for health strategy for the health sector: 2012/13–2016/17.

[17] McIntyre D, Thiede M, Nkosi M, Mutyambizi V, Castillo-Riquelme M, Gilson L (2007). SHIELD work package 1 report: a critical analysis of the current South African Health System.

[18] Econex (2010). NHI Note 7: updated GP and specialist numbers for South Africa.

[19] McIntyre D, Goudge J, Harris B, Nxumalo N, Nkosi M (2009). Prerequisites for national health insurance in South Africa: results of a national household survey. SAMJ.

[20] Ataguba J, Akazili J, McIntyre D (2011). Socioeconomic-related health inequality in South Africa: evidence from General Household Surveys. Int J Equity Health.

[21] Statistics South Africa (2013). Statistical release.

[22] Colborn R, Kane-Berman J, Hermann A, Van Niekerk J (1996). Limited private practice at academic hospitals - an ‘in-house’ group practice. SAMJ.

[23] Hoffenberg R (1997). Personal view: the future of medical education in South Africa. Br Med J.

[24] Public Service Commission (2004). Remunerative work outside the public service: an investigation undertaken in the Gauteng Provincial Health Sector.

[25] Wadee H, Khan F (2007). Human resources for health. South African Health Review 2007: The role of the private sector within the South African health system.

[26] Ashmore J (2013). ‘Going private’: A qualitative comparison of medical specialists’ job satisfaction in the public and private sectors of South Africa. Hum Resour Heal.

[27] Wilson F (1999). Organizational behavior: a critical introduction.

[28] Mbindyo P, Blaauw D, Gilson L, English M (2009). Developing a tool to measure health worker motivation in district hospitals in Kenya. Hum Resour Heal.

[29] Mathauer I, Imhoff I (2006). Health worker motivation in Africa: the role of non-financial incentives and human resource management tools. Hum Resour Heal.

[30] Franco L, Bennett S, Kanfer R (2002). Health sector reform and public sector health worker motivation: a conceptual framework. Soc Sci Med.

[31] Tzeng H (2002). The influence of nurses’ working motivation and job satisfaction on intention to quit an empirical investigation in Taiwan. Int J Nurs Stud.

[32] Lambert E, Hogan N, Barton S (2001). The impact of job satisfaction on turnover intent: a test of a structural measurement model using a national sample of workers. Soc Sci J.

[33] Locke E, Dunnette M, Hough L (1976). The nature and causes of job satisfaction. The handbook of industrial and organizational psychology.

[34] Spector P (1997). Job satisfaction: application, assessment, causes and consequences.

[35] Bluedorn A (1982). A unified model of turnover from organizations. Hum Relations.

[36] Tett R, Meyer J (1993). Job satisfaction, organizational commitment, turnover intention, and turnover: path analyses based on meta-analytic findings. Pers Psychol.

[37] Porter L, Steers R, Mowday R (1974). Organizational commitment, job satisfaction, and turnover among psychiatric technicians. J Appl Psychol.

[38] Mobley W (1977). Intermediate linkages in the relationship between job satisfaction and employee turnover. J Appl Psychol.

[39] Humphrey C, Russell J (2004). Motivation and values of hospital consultants in South-East England who work in the National Health Service and do private practice. Soc Sci Med.

[40] McClintock W, Taylor N (2004). Analysis of interviews with people holding multiple jobs in the health sector: multiple job holding in New Zealand.

[41] Glesne C (1999). Becoming qualitative researchers.

[42] Lincoln Y, Guba E (1985). Naturalistic inquiry.

[43] Rensburg D Van (2004). Health and health care in South Africa.

[44] Erasmus E, Blaauw D, Gilson L (2009). Private wards in public hospitals in South Africa: The policy context and models of operation.

[45] Bian Y, Sun Q, Jan S, Yu J, Meng Q (2003). Dual practice by public health providers in Shandong and Sichuan Provinces, in China. HEFP working paper 07/03.

