# Supplementary material for: Conceptualizing the impacts of dual practice on the retention of public sector specialists - evidence from South Africa
Source: Hum Resour Health. 2015 Jan 19;13(1):3. doi: 10.1186/1478-4491-13-3 (PMC4320565; doi:10.1186/1478-4491-13-3)
Supplement: Supplementary file 1 — Additional file 1: Hierarchy of South African hospital doctors. (PDF 46 KB) [file 12960_2014_471_MOESM1_ESM.pdf]

Additional file 1: Hierarchy of South African hospital doctors

The list below is in descending order, with doctors higher up responsible for supervising doctors lower down. This hierarchy is more rigid in public hospitals, whereas in private hospitals there tends to be fewer layers of doctors, and doctors may in fact work alone.

- **Chief Specialist and Principal Specialist** – the most senior clinical position for doctors to hold. Heads of Department (HODs) are often Chief or Principal Specialists.
- **Senior Specialist** – junior specialists may be promoted to senior specialist level, which implies more pay and greater responsibility. Note this is different from 'sub-specialists', who have further specialized in a sub-discipline (see below), e.g. neonatology within the field of pediatrics, or orthopedic surgery in the field of surgery. Sub-specialists are not necessarily senior specialists.
- **Junior Specialist** – doctors who have passed their registrar exams (see below), and are generally recently-qualified in a specialty such as anesthesiology, medicine, obstetrics and gynecology, psychiatry, or surgery.
- **Registrar** – a Medical Officer (see below) who is undertaking specialist training – which is a four year appointment. It is also possible to sub-specialize, in which case one is a senior (as opposed to junior) registrar – generally a two year undertaking.
- **Medical Officer (MO)** – MOs are essentially General Practitioners (GPs) working in hospitals. In other words, these are doctors who have completed their mandatory internship and community service, and who are qualified to practice general medicine but have not specialized. Some doctors never go on to specialize, though they may only work in specific fields. There are various ranks of MO, including junior MOs and senior or Principal MOs (PMOs). MOs may take on similar duties to specialists, but they are supervised by specialists. MOs are known as 'Senior House Officers' (SHOs) in the UK.
- **Community Service Officer (CSO)** – doctors who have finished their internship (below) must work for an additional year in an area decided by the South African government – often a rural location. Doctors are allowed to submit a list of preferences, which is taken into consideration along with staffing needs. The position is paid.
- **Intern** – after graduating medical school, South African doctors are required to undertake an internship to build hands-on experience. Location is chosen similarly to CSO appointments (see above) and the position is paid.
