# Supplementary material for: Conceptualizing the impacts of dual practice on the retention of public sector specialists - evidence from South Africa
Source: Hum Resour Health. 2015 Jan 19;13(1):3. doi: 10.1186/1478-4491-13-3 (PMC4320565; doi:10.1186/1478-4491-13-3)
Supplement: Supplementary file 2 — Additional file 2: Summary of 2009/10 RWOPS regulations. (PDF 142 KB) [file 12960_2014_471_MOESM2_ESM.pdf]

Additional file 2: Summary of 2009/10 RWOPS regulations

RWOPS regulations can be summarized for current purposes as follows. This is a summary of the most recent policy documents obtained at the time of fieldwork in H1 in 2009, including an internal circular, a copy of the policy document itself, and an example of the application form and work plan that applicants must complete to apply for RWOPS. The details of the policy below were checked and re-checked with policymakers and managers, and the information presented is accurate to the authors' best knowledge.

- **All *permanent* staff, whether full-time or part-time, must apply for RWOPS** if they wish to engage in *any* extra remunerated work not stipulated in their contract.
- **RWOPS may be performed only *after* permission is granted**.
- **RWOPS must be re-applied for every 12 months.** In some hospitals RWOPS permission may roll over.
    - **Applications must be approved by supervisors, the institutional head and the 'RWOPS committee'** (comprising members of the Clinical Management team, HODs, an HR representative, and a representative from a university, if applicable). Technically, pre-specialist staff in training may also apply (e.g. registrars, though no applications of this sort were granted at H1), but they must also gain approval from their educational institutions. Staff working at other provincial health institutions must gain approval from both institutions, and both must share the responsibility of monitoring.
- **RWOPS may in no way jeopardize the goals of the hospital and hospital departments.** Applicants must explain in writing how their RWOPS are of benefit to the Province and community.
- **Staff may not work more than 72 hours per week in total,** including on-call arrangements and RWOPS itself. A work plan must be included in each application that shows on a daily basis, for a four week period, how hours worked break down.
    - For a full-time permanent specialist employee, this would include needing to account for the following each week: 40 hours of 'normal working' time, *which may only be worked between the hours of 7am-7pm*; 16 hours of mandatory commuted overtime; leaving a maximum of 16 hours for RWOPS, depending on the on-call arrangements of the individual.
    - As such: **RWOPS may be performed at any time, so long as 'normal working' time, commuted overtime and on-call are still performed separately in full**, and as agreed in the work plan. [in H1 there was a lot of confusion about this rule].

- **Heads of Department are charged with the responsibility of ensuring procedures for monitoring are followed correctly, including that working hours stipulations are rostered.** Duty rosters must be maintained by HODs for purposes of leaving an auditable 'paper trail'. The HR department is charged with ensuring applications are filled out correctly. The CEO or hospital head is also ultimately responsible for the policy's application, and at university hospitals, the head of the medical school carries joint responsibility.
- **RWOPS can be withdrawn for any staff member with one month's (previously two months) notice, should the 'operational requirements' of the institution, department or directorate change.** Should any of the procedures in the policy not be fully followed, RWOPS can also be withdrawn immediately.
- **Should any staff member be found to perform RWOPS without permission, or should they perform RWOPS before such permission is forthcoming, they are liable to have their earnings from private work confiscated, as stipulated in the 1994 Public Service Act.**
- **Diversion of time from required public sector activities is legally considered as fraud, and RWOPS without appropriate permission is breach of contract.** RWOPS applicants acknowledge that may be audited to check for discrepancies.
